# Clinical application of metagenomic next-generation sequencing in non-immunocompromised patients with severe pneumonia supported by veno-venous extracorporeal membrane oxygenation

**DOI:** 10.3389/fcimb.2023.1269853

**Published:** 2023-10-13

**Authors:** Xing-Xing Li, Cheng-Zhi Niu, Yang-Chao Zhao, Guo-Wei Fu, Hui Zhao, Ming-Jun Huang, Jun Li

**Affiliations:** ^1^ Department of Extracorporeal Life Support Center, Department of Cardiac Surgery, The First Affiliated Hospital of Zhengzhou University, Zhengzhou, China; ^2^ Information Center, The First Affiliated Hospital of Zhengzhou University, Zhengzhou, China

**Keywords:** metagenomic next-generation sequencing, conventional culture, extracorporeal membrane oxygenation, non-immunocompromised patients, antibiotic treatment

## Abstract

**Objectives:**

This study aims to explore the pathogen-detected effect of mNGS technology and its clinical application in non-immunocompromised patients with severe pneumonia supported by vv-ECMO.

**Methods:**

A retrospective analysis was conducted on a cohort of 50 non-immunocompromised patients who received vv-ECMO support for severe pneumonia between January 2016 and December 2022. These patients were divided into two groups based on their discharge outcomes: the deterioration group (Group D), which included 31 cases, and the improvement group (Group I), consisting of 19 cases. Baseline characteristics and clinical data were collected and analyzed.

**Results:**

Among the 50 patients enrolled, Group D exhibited a higher prevalence of male patients (80.6% *vs*. 52.6%, p < 0.05), more smokers (54.8% *vs*. 21.1%, p < 0.05), and were older than those in Group I (55.16 ± 16.34 years *vs*. 42.32 ± 19.65 years, p < 0.05). Out of the 64 samples subjected to mNGS detection, 55 (85.9%) yielded positive results, with a positivity rate of 83.7% (36/43) in Group D and 90.5% (19/21) in Group I. By contrast, the positive rate through traditional culture stood at 64.9% (74/114). Among the 54 samples that underwent both culture and mNGS testing, 23 (42.6%) displayed consistent pathogen identification, 13 (24.1%) exhibited partial consistency, and 18 (33.3%) showed complete inconsistency. Among the last cases with complete inconsistency, 14 (77.8%) were culture-negative, while two (11.1%) were mNGS-negative, and the remaining two (11.1%) presented mismatches. Remarkably, mNGS surpassed traditional culture in pathogen identification (65 strains *vs*. 23 strains). Within these 65 strains, 56 were found in Group D, 26 in Group I, and 17 were overlapping strains. Interestingly, a diverse array of G+ bacteria, fungi, viruses, and special pathogens were exclusive to Group D. Furthermore, *Acinetobacter baumannii, Pseudomonas aeruginosa, and Klebsiella pneumoniae* were more prevalent in Group D compared to Group I. Importantly, mNGS prompted antibiotic treatment adjustments in 26 patients (52.0%).

**Conclusions:**

Compared with the conventional culture, mNGS demonstrated a higher positive rate, and emerges as a promising method for identifying mixed pathogens in non-immunodeficient patients with severe pneumonia supported by vv-ECMO. However, it is crucial to combine the interpretation of mNGS data with clinical information and traditional culture results for a comprehensive assessment.

## 1 Introduction

Severe pneumonia stands as a pressing health concern, marked by heightened short and long-term mortality rates (Bein et al., 2018; Sangla et al., 2020). This illness can manifest as community-acquired pneumonia (CAP), hospital-acquired pneumonia (HAP), or ventilator-associated pneumonia (VAP). Identifying the etiology of severe pneumonia can be particularly challenging in patients with prior antibiotic exposure or mechanical ventilation. Timely and accurate administration of antimicrobial therapy plays a pivotal role in optimizing outcomes among critically ill patients (Kalil et al., 2016; Torres et al., 2017; Metlay et al., 2019).

Veno-venous extracorporeal membrane oxygenation (vv-ECMO) has emerged as a life-support technique gaining traction in critically ill patients grappling with reversible refractory respiratory failure. The timely and precise implementation of ECMO has been proven to be a significant enhancement in survival rates and overall prognosis (Oh et al., 2021; Kochanek et al., 2022). However, when considering invasive procedures like continuous kidney replacement therapy (CRRT), deep vein puncture, tracheal intubation, and even ECMO intubation, the potential for infection should not be underestimated, these procedures can increase patients’ vulnerability to infections(Kühn et al., 2020). The decision to initiate vv-ECMO is primarily made in patients who have already undergone antibiotic therapy and mechanical ventilation, and the occurrence of HAP and VAP can further complicated antimicrobial therapy. Additionally, nosocomial infections occurring in vv-ECMO patients are associated with elevated mortality risk (Grasselli et al., 2017).

Presently, various methods for pneumonia etiological detection, encompassing morphological examination, isolation culture, and immunological techniques, exhibit distinct merits (Mazloomirad et al., 2021). However, conventional approaches have limitations concerning the time required for detection, positive identification rates, and specificity, particularly in severe infection scenarios. Metagenomic Next Generation Sequencing (mNGS), also known as high-throughput sequencing, presents several advantages, including shorter turnaround times and enhanced accuracy in pathogen identification (Lu et al., 2020). This technique can be applied to an array of specimen types, including bronchoalveolar lavage fluid (BALF), sputum, blood, pleural effusion, and tissue, and requires only a modest quantity of DNA/RNA extraction for efficient pathogen detection and identification (Han et al., 2019; Miller and Chiu, 2021). Moreover, mNGS allows for quantitative or semi-quantitative assessment of organism concentration in a sample through sequencing reads, which proves particularly advantageous in detecting multiple microbial strains and rare pathogens (Salipante et al., 2014). Importantly, mNGS demonstrates relatively lower susceptibility to the effects of prior antibiotic exposure compared to conventional methods (Chiu and Miller, 2019; Huang et al., 2020; Tang et al., 2021). Furthermore, the treatment adjustments guided by mNGS has shown rapid symptom alleviation, improved lung imaging, reduced hospitalization durations, and mitigation of antibiotic-related adverse reactions (Wang et al., 2020; Sun et al., 2021; Zhou et al., 2021).

In this study, we conducted a comprehensive review of non-immunocompromised patients with severe pneumonia who underwent vv-ECMO at the First Affiliated Hospital of Zhengzhou University. Our investigation assessed the value of mNGS technology in both identifying the etiology of pneumonia and guiding clinical strategy.

## 2 Materials and methods

### 2.1 Study population and design

We conducted a retrospective study involving non-immunocompromised patients who underwent vv-ECMO support for severe pneumonia at the First Affiliated Hospital of Zhengzhou University between January 2016 and December 2022. The inclusion criteria encompassed the following aspects: 1) Patients with severe pneumonia necessitating ECMO treatment (duration exceeding 24 hours) (Tonna et al., 2021); 2) Patients who underwent both mNGS and traditional culture at least once. The exclusion criteria included: 1) Age below 18 years; 2) Pregnancy; 3) Incomplete data; and 4) Immunocompromised patients. The category of immunocompromised patients encompassed those with acquired immunodeficiency syndrome (AIDS/HIV), individuals undergoing continuous (>3 months) or high-dose (>0.5 mg/kg/day) corticosteroid or immunosuppressive therapy, recipients of solid-organ transplants, individuals with rheumatic autoimmune diseases, patients with solid-organ malignancies necessitating chemotherapy within the last 5 years, individuals with a history of hematological malignancies, or those with primary immune deficiencies.

Throughout study period, a total of 151 non-immunocompromised patients with severe pneumonia required vv-ECMO intervention. Ultimately, 50 patients, comprising 35 men and 15 women, were successfully enrolled and stratified into two distinct groups based on their discharge status: the deterioration group (Group D) with 31 patients and the improvement group (Group I) with 19 patients.

Blood samples or BALF were systematically collected and routinely cultured from all enrolled patients prior to and within 24 hours after vv-ECMO initiation. Additionally, independent mNGS testing was conducted within the same 24-hour window following vv-ECMO assistance. This allowed us to analyze baseline characteristics and clinical data, including pathogens and antibiotic regimens, among non-immunocompromised patients with severe pneumonia receiving vv-ECMO support. Relevant clinical data of ECMO patients were sourced from the electronic medical record system. Patient condition evaluation included the Murray Lung Injury Score, Sequential Organ Failure Assessment (SOFA) score, and Acute Physiology and Chronic Health Evaluation II (APACHE II) score.

### 2.2 ECMO application

All enrolled patients received vv-ECMO support for refractory reversible respiratory failure due to severe pneumonia, in alignment with the indications and contraindications set forth by the Extracorporeal Life Support Organization (ELSO) 2021 guidelines (Tonna et al., 2021). Each patient opted for percutaneous venous catheterization through the femoral vein-internal jugular vein access. The employed ECMO system kit encompassed a centrifugal pump, an oxygenator, and their respective connecting pipelines. Specifically, the centrifugal pump utilized the Rotaflow pump head and controller provided by either Medtronic or MAQUET. As for the oxygenator, options catering to adults from both Medtronic and MAQUET were taken into consideration. The pipeline configuration encompassed ECMO-specific tubing alongside standard PVC tubing, with arteriovenous thin-walled cannulae serving as an integral component. The management of the ECMO procedure adhered to the following key principles:

#### 2.2.1 Mechanical ventilation management

The primary goal was to minimize the risk of ventilator-associated lung injury while concurrently fostering alveolar recruitment.

#### 2.2.2 Anticoagulation

Unfractionated heparin was administered to sustain an activated coagulation time (ACT) that was 1.5 times the upper limit of normal, alongside an activated partial thromboplastin time (APTT) maintained within the range of 40 to 55 seconds.

#### 2.2.3 Flow management

Flow adjustments were orchestrated in accordance with the patient’s monitoring indicators, striving to uphold blood oxygen saturation (SaO_2_) within the range of 85% to 95% and maintaining a PaO_2_ level surpassing 60 mmHg. In specific respiratory support scenarios (FiO_2_ < 50%, positive end-expiratory pressure (PEEP) ≤ 10 cmH_2_O, peak airway pressure < 30 cmH_2_O), the vv-ECMO pump flow was regulated to 2 to 3 L/min and the vv-ECMO airflow was temporarily suspended for a monitoring period of 2 to 4 hours. If SaO_2_ exceeded 95% and PaCO_2_ fell below 50 mmHg, consideration could be given to the weaning of vv-ECMO.

### 2.3 The mNGS procedures

Samples were collected from infected patients within a time window of 24 hours from the initiation of ECMO support. Subsequently, these samples earmarked for mNGS underwent a multi-step process managed by specialized testing company. This process included nucleic acid extraction, library construction, high-throughput sequencing, bioinformatics analysis, result presentation, and the interpretation of pathogen-related data.

#### 2.3.1 Nucleic acid extraction

In a systematic batch process, 300μl of blood samples and 600μl of preprocessed BALF samples were subjected to nucleic acid (both DNA and RNA) extraction.

#### 2.3.2 DNA library construction

DNA libraries were meticulously constructed through a sequence of steps encompassing DNA fragmentation, end-repair, adapter ligation, and PCR amplification. Subsequently, these libraries underwent a quality assessment using an Agilent 2100 bioanalyzer and ABI StepOnePlus Realtime PCR System. Only qualified libraries advanced to the next stages.

#### 2.3.3 Sequencing

The qualified DNA libraries were subjected to sequencing on the NextSeq 550Dx platform (Illumina), employing a sequencing read length of 75-bp.

#### 2.3.4 Metagenomics sequencing

For metagenomic sequencing, the Illumina NextSeq 550 sequencer was employed. Each sequencing batch accommodated 15 to 20 samples, including a negative control. An internal reference derived from Arabidopsis thaliana was provided by the sequencing manufacturers.

#### 2.3.5 Data processing and analysis

Through a meticulous process of data curation, high-quality sequencing data were generated by eliminating low-quality and short reads (<35 bp in length). The retained reads were then rigorously aligned to both pathogen species (SDSMRN) and pathogen genus (SDSMRNG). This alignment process yielded a curated list of microorganisms.

#### 2.3.6 Comparison and validation

The curated list of microorganisms was subsequently cross-referenced with an internal background database housing microorganisms present in over 50% of samples from the laboratory over the past three months. Any microorganisms suspected to be background contaminants were excluded from further analysis.

#### 2.3.7 Identification of suspected pathogens

Microorganisms meeting specific criteria were considered suspected pathogens. Those with SDSMRN > 50 and at least three times higher abundance compared to the control group were deemed as such. For suspected pathogens with SDSMRN < 50, they needed to exhibit at least a fivefold increase in abundance compared to the control group to be considered.

### 2.4 Ethics statement

The studies involving human participants were reviewed and approved by the Ethics Committee of the First Affiliated Hospital of Zhengzhou University (2022-KY-0272).

### 2.5 Statistical analysis

All collected data were statistically analyzed using SPSS 21.0 (IBM Corp., Armonk, NY, USA). Continuous variables were expressed as mean ± standard deviation and compared using unpaired t-test. If continuous variables were skewed, they were expressed as quartiles and compared using the Mann-Whitney U test. Categorical variables were expressed as frequency (composition ratio) and compared using Pearson’s χ test or Fisher’s exact test. A significance level of p < 0.05 was considered statistically significant.

## 3 Results

### 3.1 Characteristics and laboratory examinations of ECMO patients

**Figure 1:**
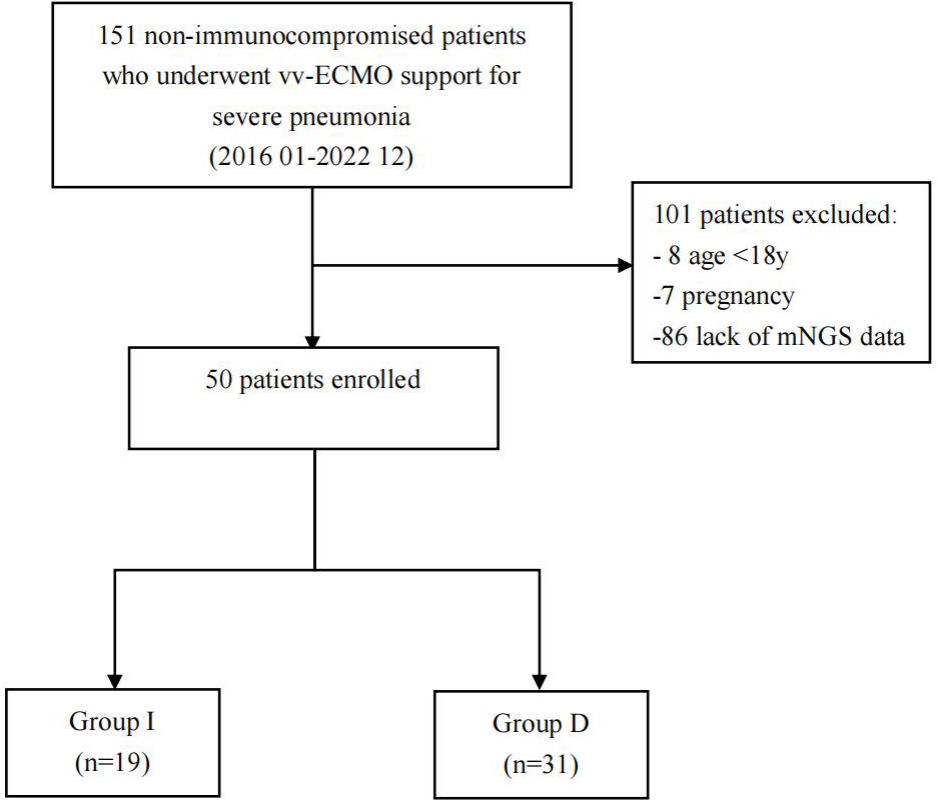
Flowchart of analyzed patients. VV-ECMO, veno-venous extracorporeal membrane oxygenation; mNGS, metagenomic next-generation sequencing; Group D, deterioration group; Group I, improvement group.

The experiment’s flowchart is presented in **Figure 1**. **Table 1** outlines the characteristics of enrolled patients, revealing that 70.0% of them were male. Notably, there were no substantial differences between the two groups in terms of various parameters. These encompassed body mass index (BMI), baseline vital sign data (heart rate, respiratory rate, and mean arterial pressure), and comorbidities such as chronic obstructive pulmonary disease (COPD), interstitial lung disease (ILD), hypertension, type 2 diabetes, and coronary heart disease. Additionally, no significant disparities were observed in pre-ECMO system scores, including the Murray Lung Injury Score and sequential organ failure assessment (SOFA) score. Similarly, the system scores 24 hours after ECMO initiation (SOFA scores and APACHE II scores), exhibited no noteworthy differences between the two groups (all p > 0.05, **Table 1**).

**Table 1:** Baseline characteristics of non-immunocompromised patients with severe pneumonia supported by vv-ECMO.

Variables	Group D (n=31)	Group I (n=19)	p-value
**Male, n (%)**	25(80.6%)	10(52.6%)	0.036
**Age (year)**	55.16±16.34	42.32±19.65	0.016
**BMI (kg/m2)**	24.25±3.32	22.82±3.54	0.158
**Smoking, n (%)**	17(54.8%)	4(21.1%)	0.019
Comorbidities, n (%)			
COPD	6(19.4%)	5(26.3%)	0.822
ILD	5(16.1%)	1(5.3%)	0.485
Hypertension	5(16.1%)	6(31.6%)	0.353
Type 2 diabetes	2(6.5%)	0(0%)	0.519
Coronary heart disease	5(16.1%)	1(5.3%)	0.484
**Murray Lung Injury Score**	3.19±0.44	3.07±0.42	0.332
**SOFA scores (before ECMO assistance)**	12.55±4.15	14.32±6.43	0.294
**SOFA scores (24 h of ECMO assistance)**	13.06±4.55	13.74±6.36	0.665
**APACHE II scores (before ECMO assistance)**	20.13±6.52	15.63±5.19	0.014
**APACHE II scores (24 h of ECMO assistance)**	15.32±4.97	12.42±6.81	0.089
**Respiratory rate**	27.00±9.2	24.68±7.21	0.355
**Heart rate**	114.03±22.64	112.58±18.62	0.815
**Systolic pressure (mmHg)**	119.74±17.45	118.63±19.20	0.834
**Diastolic pressure (mmHg)**	75.48±15.51	69.47±13.47	0.169
**Mean arterial pressure (mmHg)**	90.16±14.83	85.84±14.66	0.321
**PaO2/FiO2 before ECMO**	51(43.75-60.15)	55.8(43.7-66.55)	0.322
**PEEP before ECMO (cmH2O)**	9.29±3.75	10.32±3.38	0.336
**PEEP after ECMO (cmH2O)**	8.87±3.11	9.05±2.76	0.835
**Vasoactive drug, n (%)**	15(48.4%)	7(36.8%)	0.425
**Pneumothorax, n (%)**	1(3.2%)	1(5.3%)	1.000
**Prone position ventilation, n (%)**	18(58.1%)	10(52.6%)	0.707
**MODS, n (%)**	17(54.8%)	7(36.8%)	0.216
**CRRT, n (%)**	14(45.2%)	4(21.1%)	0.085
ECMO related complications, n (%)			
Pulmonary hemorrhage	4(12.9%)	0(0%)	0.273
Coagulation dysfunction	11(35.5%)	3(15.8%)	0.132
DIC	1(3.2%)	1(5.3%)	1.000
Cerebral infarction	1(3.2%)	0(0%)	1.000
Cerebral hemorrhage	3(9.7%)	2(10.5%)	1.000
Limb complications	18(58.1%)	7(36.8%)	0.145
Renal insufficiency	14(45.2%)	6(31.6%)	0.341
Hepatic insufficiency	14(45.2%)	6(31.6%)	0.341
**Mechanical ventilation time before ECMO (h)**	22(4-71)	9(2-25)	0.280
**Mechanical ventilation time (h)**	276(193-544.5)	273(178.5-416)	0.317
**Duration of ECMO (h)**	240(161.75-339)	166(120-211)	0.016
**ICU stay time (days)**	15(10.5-22.19)	19(13-24.5)	0.078
**Hospital stay time (days)**	17.5±7.87	26.95±9.87	0.000

vv-ECMO, veno-venous extracorporeal membrane oxygenation; Group D, deterioration group; Group I, improvement group; BMI, body mass index; COPD, chronic obstructive pulmonary disease; ILD, interstitial lung disease; SOFA, sequential organ failure assessment score; PEEP, positive end-expiratory pressure; MODS, multiple organ dysfunction syndrome; CRRT, continuous renal replacement therapy; DIC, disseminated intravascular coagulation; ICU, intensive care unit.

The data are shown as mean ± SD, median (interquartile 25–75), or n (percentage).

Likewise, there were no notable discrepancies between the two groups in parameters such as PaO2/FiO2 ratio, positive end-expiratory pressure (PEEP), mechanical ventilation duration before and after ECMO assistance, length of stay in the intensive care unit (ICU), percentages of vasoactive drug usage, occurrences of complications like pneumothorax, prone position ventilation, multiple organ dysfunction syndrome (MODS), continuous renal replacement therapy (CRRT), and ECMO-related complications including pulmonary hemorrhage, coagulation dysfunction, disseminated intravascular coagulation (DIC), cerebral infarction, cerebral hemorrhage, limb complications, renal insufficiency, and hepatic insufficiency (all p > 0.05, **Table 1**).

However, certain distinctions emerged when examining the Group D. Notably, this group had a higher proportion of males (80.6% *vs*. 52.6%, p < 0.05, **Table 1**) and a greater prevalence of smoking (54.8% *vs*. 21.1%, p < 0.05, **Table 1**). Moreover, individuals in Group D were significantly older than those in Group I (55.16 ± 16.34 years *vs*. 42.32 ± 19.65 years, p < 0.05, **Table 1**). Importantly, Group D exhibited higher APACHE II scores before ECMO initiation (20.13 ± 6.52 *vs*. 15.63 ± 5.19, p < 0.05, **Table 1**), indicating a more severe condition. Furthermore, Group D had a notably extended duration of ECMO support [240 (161.75-339.00) hours *vs*. 166 (120-211) hours, p < 0.05, **Table 1**], while their hospital stay was significantly shorter compared to Group I (17.5 ± 7.87 days *vs*. 26.95 ± 9.87 days, p < 0.001, **Table 1**), indicating patients in the deterioration group were considered to have deteriorated earlier and were automatically discharged from the hospital.

In comparison to Group I, Group D exhibited distinct biochemical profiles throughout different phases of the experiment (**Table 2**). Logistic regression analysis showed that lactate level at ECMO weaning was an independent risk factor for deterioration in patients.

**Table 2:** Comparison of laboratory examinations at three different time points.

Variables	24 h before ECMO		24 h within ECMO		ECMO weaned	
	Group D (n=31)	Group I (n=19)	Group D (n=31)	Group I (n=19)	Group D (n=31)	Group I (n=19)
**WBC**^ (*109/L)	11.98±6.5	8.9±4.75	13.12±7.45	9.27±4.67	16.11±11.87	12.55±4.23
**Neutrophils**^ (*10^9^/L)	11.02±6.19	8.06±4.71	11.94±6.88	7.69±4.88	14.62±11.16	10.74±3.85
**Lymphocyte** (*10^9^/L)	0.48(0.14-1.01)	0.45(0.34-0.75)	0.42(0.26-0.79)	0.62(0.37-0.71)	0.46(0.31-1.12)	0.85(0.66-1.07)
**Hb** ^^&^(g/L)	113±23.65	108.01±24.47	98.03±20.51	112.88±23.82	89.41±12.77	98.53±12.22
**PLT** ^&^(*10^9^/L)	171.32±88.95	135.84±76.48	132.71±78.51	119.47±52.3	76.74±68.97	127.74±60.24
**ALT** ^#^ (U/L)	34(19.5-56.5)	44(27.5-106)	39(25.5-88)	30(18-47)	38(26-80.5)	40(22-56)
**AST** ^&^(U/L)	43(29.5-86.5)	68(50.75-169.5)	60(39.5-99)	53(31.5-119.5)	52(40.5-124.5)	40(28.5-63.5)
**Albumin** (g/L)	30.85±6.53	30.7±9.63	30.77±8.67	33.28±9.85	36.7±10.08	38.65±7.75
**Globulin** (g/L)	28.6±7.2	26.66±5.44	27.4±7.6	23.59±6.24	30.26±10.75	26.31±8.31
**TB** ^&^ (mmol/L)	15.2(10.9-24.9)	14.8(5.55-27.45)	15(11.55-35.95)	20.3(10.14-29.82)	48.5(20.99-118.7)	21(15-30.9)
**DB** ^&^(mmol/L)	9.4(5.6-15.2)	6.6(3.8-20.15)	8.5(5.8-28.2)	12.4(5.75-20)	35.4(13.8-91)	10.7(6.905-16.95)
**LDH** ^&^ (U/L)	729(595-1005.5)	785(382-1086)	679(442-1188.5)	506(334-769)	971.5(578.5-1998.5)	454(359.5-559)
**BUN** ^#^^^&^(mmol/L)	11.8(5.76-15.75)	6.79(6.12-8.91)	11.3(8.35-16.05)	8(6.25-9.45)	16.9(13.6-24.06)	9.5(7.2-15.6)
**Cr** ^&^ (μmol/L)	65(55.5-87)	64(53.5-103)	65(55.5-105.35)	67.2(63-93.55)	96(71-187.5)	66(44-76.95)
**eGFR** (ml/min)	85.83±40.52	99.62±37.73	81.74±31.10	93.70±30.74	72.4±37.62	92.47±35.17
**PT** ^&^ (s)	13.68±2.71	19.02±18.24	15.15±3.28	14.06±2.89	15.67±3.85	13.25±3.86
**INR** ^&^	1.2±0.24	1.21±0.43	1.37±0.29	1.28±0.27	1.41±0.35	1.21±0.35
**APTT** (s)	32.87±8.51	34.05±8.61	55.32±31.97	56.16±23.21	51.17±19.92	44.66±10.2
**Fibrinogen** (g/L)	4.25±1.79	3.6±2.28	3.05±1.23	3.22±1.32	3.22±1.68	2.96±1.09
**D-dimer** (mg/L)	1.34(0.47-4.16)	2.65(0.475-8.85)	2.71(1.22-9.87)	1.925(0.71-3.75)	2.38(1.16-6.97)	2.39(1.03-5.14)
**FDP**(μg/mL)	13.34(5.39-48.94)	19.31(4.9-51.85)	19.8(6.54-76.4)	11.85(5.87-42.57)	14.2(9.28-50.68)	18.12(13.24-31.18)
**NT-pro-BNP** ^#^^^&^(pg/ml)	1514(484.05-5066.5)	422(180.28-1895.5)	2384.38(663.49-4449)	277.1(226.06-1901.5)	2074(790.1-3645.41)	306.8(190.5-786.5)
**PCT** ^&^(ng/ml)	1.33(0.354-2.13)	1.5(0.5795-4.69)	2.7(0.94-8.48)	2.01(1.2-5.05)	3.348(1.31-13.38)	0.61(0.22-1.14)
**CRP** ^&^ (mg/L)	150(95.4-189.22)	85.8(37.27-113.7)	135.2(62.39-184.35)	69.28(38.51-119.18)	114.07(59.55-186.24)	45(11.85-92.77)
**ESR** ^#^ (mm/h)	48.5(34.95-75.4)	29(16.05-50.6)	30(17.75-41.25)	24.4(8.95-35.3)	38.6(25.55-70.5)	26.4(18.35-49.9)
**PH** ^&^	7.34±0.12	7.31±0.14	7.43±0.1	7.42±0.12	7.34±0.15	7.45±0.05
**PaO2** (mmHg)	54.49±22.35	59.37±22.29	81.1±21.73	85.15±20.11	99.27±82.53	107.9±43.3
**PaCO2** (mmHg)	46.12±12.49	47.56±15.48	37.01±8.14	36.08±10.31	42.19±9.4	41.71±7.58
**Lactate** ^&^ (mmol/L)	2.98±2.81	2.65±2.3	2.97±2.11	2.69±2.24	6.12±6.36	1.48±0.82

Group D, deterioration group; Group I, improvement group; ECMO, extracorporeal membrane oxygenation; WBC, white blood cells; ALT, alanine aminotransferase; AST, aspartate aminotransferase; TB, total bilirubin; DB, direct bilirubin; LDH, lactic dehydrogenase; BUN, blood urea nitrogen; Cr, creatinine; eGFR, estimated glomerular filtration rate; PT, prothrombin time; INR, international normalized ratio; APTT, activated partial thromboplastin time; FDP, Fibrinogen Degradation Products; CRP, C-reactive protein; ESR, erythrocyte sedimentation rate.

The data are shown as mean ± SD, median (interquartile 25–75), or n (percentage).

^#^Statistical significance in 24 h before ECMO.

^^^Statistical significance in 24 h within ECMO.

^&^Statistical significance when ECMO was weaned.

### 3.2 Strain differences of mNGS detection and traditional culture

Among the 50 ECMO patients included in the study, a total of 64 samples were subjected to mNGS analysis. These samples encompassed 13 (20.3%) blood samples and 51 (79.7%) BALF samples. Among these, a substantial 55 (85.9%) returned positive results. Of these positive samples, 36 (83.7%) were from Group D, 19 (90.5%) were from Group I; 9 (69.2%) were blood samples, and 46 (90.2%) were BALF samples. Note that there were no statistically significant differences in the positive rate of mNGS between the two groups and the different samples (P=0.729, P=0.135).

**Figure 2:**
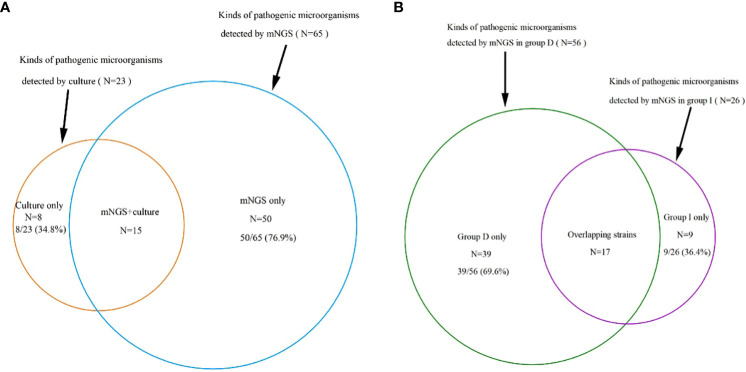
**(A)**: A total of 65 kinds of pathogenic microorganisms were detected by mNGS, 23 were cultured from conventional culture, and 15 kinds of pathogenic microorganisms were found in both mNGS and conventional culture, which accounted for 23.1% and 65.2% of the total, respectively, proving that the mNGS assay can detect more pathogenic microorganisms. **(B)**: 65 strains were detected by mNGS, including 56 in group D, 26 in group I and 17 overlapping strains. mNGS, metagenomic next-generation sequencing; Group D, deterioration group; Group I, improvement group.

In contrast, a total of 114 samples underwent traditional culture analysis, resulting in 74 (64.9%) of them being classified as positive. Even though a greater number of samples were subjected to traditional culture compared to mNGS detection, the latter method revealed a wider array of identified pathogenic microorganisms (65 strains *vs*. 23 strains) (**Figure 2A**). More specifically, among the 65 strains, 56 were identified in Group D, 26 in Group I, and 17 were found to overlap (**Figure 2B**). For a single sample mNGS detects two and more pathogens at a higher.

### 3.3 Pathogenic microorganisms

**Figure 3:**
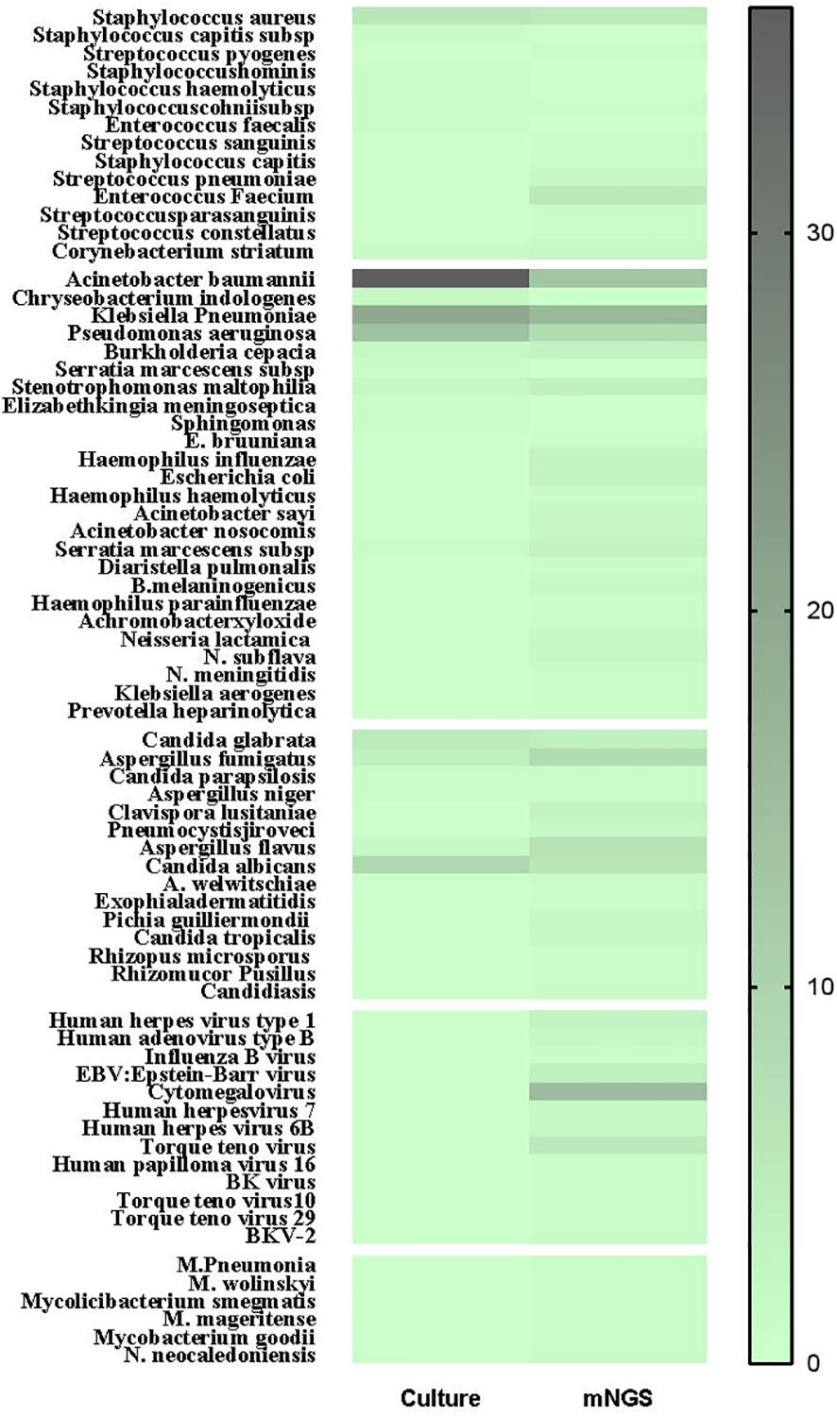
The heat map of pathogenic microorganisms details in different methods (mNGS and conventional culture). mNGS, metagenomic next-generation sequencing.

The diversity in the spectrum of pathogenic microorganisms detected by the two techniques stemmed from the fact that only 15 pathogenic microorganisms were common to both methods. These accounted for 65.2% of the organisms identified through traditional culture and 23.1% of those identified via mNGS (**Figure 2A**). Notably, the traditional culture method lacked the capacity to cultivate viruses and specialized microorganisms that mNGS could detect, as illustrated in **Figure 3**.

Within the realm of traditional culture, the most frequently detected microorganisms were *Acinetobacter baumannii*, *Klebsiella pneumoniae*, and *Pseudomonas aeruginosa*, all of which fell under the classification of Gram-negative bacteria. On the other hand, the prevalent representatives among Gram-positive bacteria, Gram-negative bacteria, and fungi were *Staphylococcus aureus*, *Acinetobacter baumannii*, and *Candida albicans*, respectively. In contrast, the organisms with the highest frequency of mNGS detection were *Klebsiella pneumoniae*, *Cytomegalovirus*, and *Acinetobacter baumannii* (**Figure 3**).

**Figure 4:**
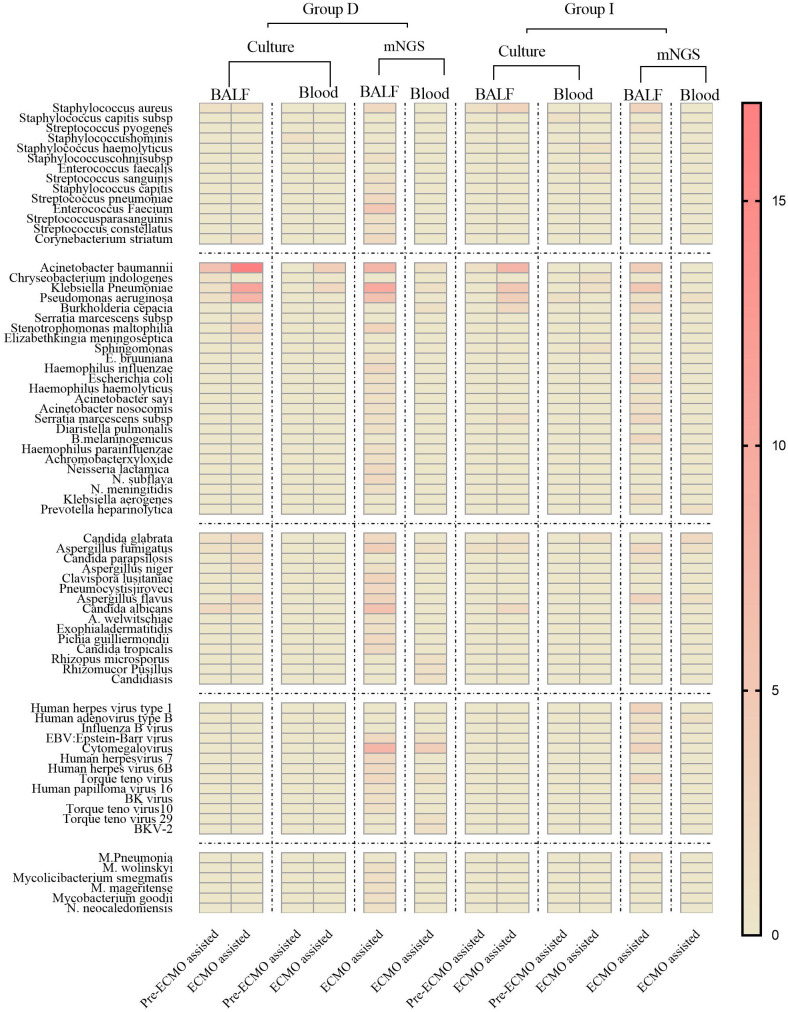
The heat map of pathogenic microorganism details from different samples (BALF and Blood) in group D and group I in different time periods (before ECMO assistance, within 48 h of ECMO assistance, after 24 h of ECMO assistance). Group D, deterioration group; Group I, improvement group; ECMO, extracorporeal membrane oxygenation; mNGS, metagenomic next-generation sequencing; BALF, bronchoalveolar lavage fluid.

Group D displayed a broader spectrum of detected pathogens, including various Gram-positive bacteria, fungi, viruses, and specific pathogens. Notably, *Acinetobacter baumannii*, *Pseudomonas aeruginosa*, and *Klebsiella pneumoniae* exhibited higher frequencies in Group D compared to Group I (**Figure 4**). **Figure 4** delved into the nuanced details of pathogenic microorganisms present in different sample types (BALF and Blood) within distinct time frames (prior to ECMO assistance and within 24 hours of ECMO assistance) for both the deterioration and improvement groups. Notably, **Figure 4** underscored how mNGS could play a pivotal role in diagnosing specific infections, encompassing mixed infections and those arising from special pathogens.

### 3.4 Comparison of mNGS results and culture results during ECMO assistance

The present study highlights the superior pathogen detection capabilities of mNGS compared to the conventional culture method. Our investigation delved into the congruence of pathogen identification outcomes yielded by both techniques during ECMO assistance. In this analysis, we categorized test results as consistent when the pathogens identified by mNGS aligned precisely with those isolated through culture. Furthermore, if mNGS unveiled more pathogens than the culture method, we deemed the results as consistently aligned. Partial consistency was considered when pathogens identified by both techniques were partially congruent. Conversely, results were classified as inconsistent when the pathogens identified by both approaches diverged entirely.

**Figure 5:**
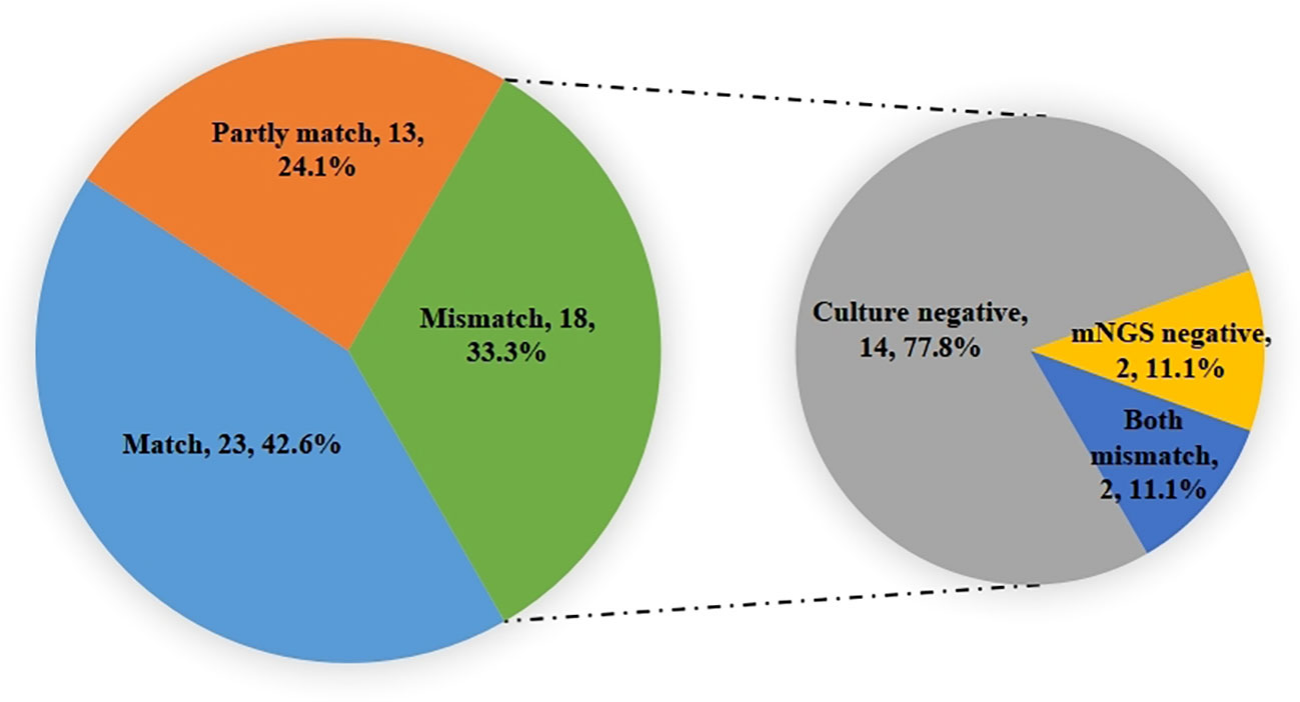
The consistent analysis comparing culture and mNGS pathogen detection during ECMO assistance. Identified pathogens 23(42.6%) were consistent, 13(24.1%) were partially consistent, and 18(33.3%) were completely inconsistent. In the inconsistent ones, 14(77.8%) were negative for the culture method, while 2(11.1%) were negative for mNGS, and 2(11.1%) were mismatched.

Among the 50 patients enrolled, a total of 54 samples underwent both culture and NGS testing during ECMO assistance. The comparison yielded intriguing results: 23 samples (42.6%) exhibited consistent pathogen identification, 13 samples (24.1%) were partially consistent, and 18 samples (33.3%) demonstrated complete inconsistency. In instances of complete inconsistency, 14 (77.8%) were negative when subjected to the culture method, while 2 (11.1%) yielded negativity when examined using mNGS. Additionally, 2 (11.1%) showcased mismatched results between the two techniques (**Figure 5**).

### 3.5 Application of mNGS in non-immunocompromised patients with severe pneumonia supported by vv-ECMO for antibiotic therapy

In our study, our focus extended to evaluating the practical implications of the assay’s findings within a clinical context. Among the participants, a notable 26 patients (52.0%) saw adjustments made to their antibiotic therapy based on mNGS results. This adjustment was observed in 17 cases (54.8%) within Group D and 9 cases (47.4%) within Group I. Despite the slightly higher percentage of antibiotic adjustments in Group D, statistical analysis revealed no significant disparity between the two groups (P=0.608). For a comprehensive view of the impact, details of the antibiotic treatment adjustments for 16 pathogens can be found in **Table 3**.

**Table 3:** Pathogens initiate targeted antimicrobial treatment after acquiring the results of mNGS.

Pathogens	Targeted Antimicrobial Treatment
Mold funli(n=2)	Amphotericin B (n=1)
	Esaconazole (n=1)
Monilia (n=3)	Fluconazole (n=1)
	Amphotericin B aerosol inhalation (Liver damage) (n=1)
	Amphotericin B (n=1)
Aspergillus(n=4)	Voriconazole (n=3)
	Voriconazole + carpofungin (n=1)
Aspergillus+Monilia (n=2)	Carpofungin(n=2)
Human herpes virus(n=4)	Ganciclovir (n=2)
	Penciclovir (n=2)
Influenza B virus(n=1)	Oseltamivir(n=1)
Mycoplasma(n=1)	Moxifloxacin(n=1)
Pneumocystis jirovecii (n=2)	TMP/SMZ(n=2)
Stenotrophomonas maltophilia (n=2)	Ceftazidime(n=1)
	TMP/SMZ(n=1)
Burkholderia cepacia(n=1)	Meropenem(n=1)
Staphylococcus aureus(n=1)	Biapenem + Linezolid(n=1)
Staphylococcuscohniisubsp(n=1)	Vancomycin(n=1)
Staphylococcus capitis(n=1)	Vancomycin(n=1)
Acinetobacter baumannii(n=2)	Cefperazone-Sulbactam(n=1)
	Piperacillin-tazobactam(n=1)
Klebsiella Pneumoniae(n=8)	Cefperazone-Sulbactam (n=2)
	Polymyxin B(n=1)
	Ceftazidime-avibactam (n=3)
	Polymyxin B(n=1)
	Meropenem(n=1)
Pseudomonas aeruginosa(n=5)	Cefperazone-Sulbactam (n=3)
	Aztrenam+Ciprofloxacin (n=1)
	Biapenem (n=1)

mNGS, metagenomic next-generation sequencing; TMP/SMZ, trimethoprim/sulfamethoxazole.

## 4 Discussion

Severe pneumonia presents a critical challenge with high mortality rates, primarily attributed to the limitations of current diagnostic techniques in pathogen identification and the escalating issue of antibiotic resistance (Costelloe et al., 2010; Currie et al., 2014; Jain et al., 2015). Consequently, timely and precise pathogen identification has emerged as a paramount necessity. In our retrospective study, we undertook an assessment of mNGS’s efficacy in pathogen detection and personalized antibiotic therapy for non-immunocompromised severe pneumonia patients receiving vv-ECMO support. Our findings substantiated that mNGS boasted a superior pathogen detection rate (85.9%) compared to traditional culture methods, identifying a broader array of pathogenic types (65 strains *vs*. 23 strains) – a remarkable achievement even in patients who had undergone prolonged antibiotic treatment before mNGS sample collection. Importantly, our study unveiled that mNGS results could contribute crucial insights for clinical decision-making regarding antibiotic therapy.

Non-immunocompromised severe pneumonia patients, despite initially normal or near-normal immune status, could develop acquired infections due to associated conditions and ECMO-induced immune dysfunction. This phenomenon underscored the significance of acquired infections during ECMO support, affecting up to 55% of patients (Al-Fares et al., 2019; Frerou et al., 2021). The pivotal role of infections in the prognosis of ECMO patients has been highlighted in earlier research (Biffi et al., 2017; Grasselli et al., 2017). While traditional diagnostic methods such as G/GM testing, bacterial culture, and quantitative polymerase chain reaction (qPCR) are commonly used, they often possess inherent limitations (Haneke et al., 2016) and can struggle to accurately detect mixed pathogens (Crotty et al., 2015). This context calls for a revolutionary diagnostic approach to enable timely and precise pathogen detection, crucial for averting uncontrolled infections that significantly influence the prognosis of critically ill patients undergoing ECMO treatment.

In addition to its heightened sensitivity, rapid detection cycles, and versatile applications, mNGS technology circumvents the need for bacterial isolation and remains unaffected by prior antibiotic usage, effectively reducing the false-negative rate (Brown et al., 2018; Carpenter et al., 2019). Recent studies even demonstrate mNGS’s capability to detect potential pathogens in samples that routine tests classify as negative, thereby enhancing diagnostic accuracy for infections (Zinter et al., 2019; Chen et al., 2021). Theoretically, mNGS has the potential to detect a comprehensive range of nucleotide sequences and pathogens, expanding the scope of infection diagnostics. Consequently, mNGS is particularly advantageous in detecting mixed pathogen infections (Wang et al., 2019). Given that patients treated with vv-ECMO have often undergone prior antibiotic treatment and mechanical ventilation, mNGS holds special value in this context. Our findings in this study echoed previous research (Cheng et al., 2019), indicating that the positive rate of traditional culture (64.9%) was lower than that of mNGS detection (85.9%). Notably, our study reinforced the robustness of mNGS in detecting a wide variety of viruses, as well as specific pathogens like *Mycoplasma pneumoniae, Mycobacterium* spp.*, and Neisseria neocaledoniensis*, reaffirming its ability to identify special pathogen infections and mixed infections.

Among the organisms frequently detected through mNGS, *Klebsiella pneumoniae, Cytomegalovirus, and Acinetobacter baumannii* emerged as predominant pathogens in non-immunocompromised severe pneumonia patients supported by vv-ECMO. Interestingly, various Gram-positive bacteria, fungi, viruses, and specific pathogens were exclusively detected in Group D, while *Acinetobacter baumannii, Pseudomonas aeruginosa, and Klebsiella pneumoniae* exhibited higher prevalence in Group D compared to Group I. These organisms are commonly associated with Hospital-Acquired Pneumonia (HAP) and Ventilator-Associated Pneumonia (VAP). Research suggests that initial viral or bacterial infections can trigger a dysregulation of cytokine and chemokine production, setting off an overactivated signaling cascade that leads to excessive immune cell recruitment and inflammatory processes. Consequently, this cascade may inflict severe lung damage, providing a gateway for pathogens to penetrate deep tissues and perpetuate viral and bacterial replication (Wilden et al., 2021). Furthermore, compromised immune responses in later stages may be attributed to pathogen-mediated inhibition of immune cells or signaling pathways, resulting in diminished pathogen clearance. These mechanisms provide insights into the increased morbidity and mortality associated with viral and bacterial co-infections (Aguilera and Lenz, 2020; Wilden et al., 2021).

The management of pneumonia remains complex due to shifting epidemiological patterns. Owing to the lack of point-of-care diagnostic tests that can accurately pinpoint microbial etiology (excluding viral pneumonia), initial antibiotic treatment often relies on empirical methods. While empirical antibiotic treatment plays a crucial role in controlling disease progression, inherent limitations persist, potentially leading to inadequate microbial antibiotic coverage and inappropriate use of broad-spectrum antibiotics. Such limitations can impact prognosis, drive drug resistance, heighten drug toxicity, and escalate treatment-related expenses (Maertens et al., 2005; Polgreen et al., 2007; Tumbarello et al., 2015). Additionally, a considerable proportion of patients (ranging from 38.0% to 60.8%) require adjustments to their antibiotic regimen due to inappropriate empirical antibiotic therapy (Alvarez-Lerma, 1996; Kollef and Ward, 1998). Amid the realm of empirical antibiotic use, early incorporation of mNGS holds the promise of targeted therapy initiation, thus enhancing clinical antibiotic management to counteract resistance. Moreover, by reducing hospital stay durations, ICU admission lengths, ventilation periods, and minimizing the occurrence of antibiotic-related complications while significantly curtailing ICU costs, mNGS can have a favorable impact on clinical outcomes (Wang et al., 2020; Zhang et al., 2020; Shen et al., 2021; Sun et al., 2021; Xie et al., 2021). In our study, 26 out of 50 patients underwent antibiotic adjustments based on mNGS results, even though there was no statistically significant difference in clinical outcomes. This could be attributed to the higher number of pathogenic microorganisms detected in Group D, where the number of antibiotic adjustment cases was higher. Moreover, mNGS testing occurred after ECMO initiation, relatively late in detection timing, possibly having less influence on patients’ conditions. Despite these limitations, the guiding role of mNGS in antibiotic treatment remains noteworthy. Further prospective studies are needed to explore whether mNGS can guide the optimization of antibiotic therapy.

While mNGS detection presents numerous benefits, traditional culture remains indispensable. In our single-center study, 34.8% of pathogens identified through traditional culture went undetected by mNGS. Therefore, a combination of traditional culture and mNGS detection for pathogen identification and drug sensitivity testing offers a more comprehensive and effective approach for guiding clinical treatment in real-world scenarios.

This study is not without limitations. The retrospective nature of our study might constrain the generalizability of the outcomes, emphasizing the necessity for broader cohort studies to validate our findings. Furthermore, not all samples underwent RNA and DNA virus detection, and the influence of mechanical ventilation on pathogen results cannot be ignored. Finally, ECMO, being an invasive procedure, could potentially affect post-ECMO treatment outcomes.

## 5 Conclusion

In conclusion, our results indicated that mNGS has a higher positive rate than the conventional culture and can be used to effectively identify mixed pathogens in non-immunocompromised patients with severe pneumonia supported by vv-ECMO, especially in those patients with negative cultures due to infections with special pathogens or prior antibiotic exposure. However, it is important to note that the interpretation of mNGS data should be complemented by clinical data and conventional culture results due to the lack of uniform diagnostic criteria.

## Data availability statement

The data that support the findings of this study have been deposited into the EMBL database with accession number PRJEB65300.

## Ethics statement

The studies involving humans were approved by the ethics committee of the First Affiliated Hospital of Zhengzhou University. The studies were conducted in accordance with the local legislation and institutional requirements. Written informed consent for participation was not required from the participants or the participants’ legal guardians/next of kin because this study is retrospective and no additional intervention should be performed on the patients.

## Author contributions

X-XL: Data curation, Formal Analysis, Software, Writing – original draft. C-ZN: Data curation, Formal Analysis, Software, Writing – original draft. Y-CZ: Conceptualization, Methodology, Supervision, Writing – review & editing. G-WF: Methodology, Validation, Writing – review & editing. HZ: Methodology, Supervision, Writing – review & editing. M-JH: Resources, Writing – review & editing. JL: Resources, Supervision, Writing – review & editing.
